# Clinical Model Predicting Presence of Diabetic Nephropathy in Renal Biopsy Performed on Patients with Diabetes

**DOI:** 10.3390/jcm15020654

**Published:** 2026-01-14

**Authors:** Maja Pieczaba, Zofia Bielenin, Marcin Ożga, Wiktor Teżyk, Krzysztof Benc, Wiktoria Pabian, Dominika Pisarek, Ewa Tabaka, Krzysztof Letachowicz, Tomasz Gołębiowski, Piotr Donizy, Agnieszka Hałoń, Andrzej Konieczny, Mirosław Banasik

**Affiliations:** 1 Clinical Department of Nephrology, Transplantation Medicine and Internal Diseases, Institute of Internal Diseases, Wroclaw Medical University, 50-556 Wroclaw, Polandandrzej.konieczny@umw.edu.pl (A.K.); miroslaw.banasik@umw.edu.pl (M.B.); 2 Faculty of Medicine, Wroclaw Medical University, 50-367 Wroclaw, Polandwiktor.tezyk@student.umw.edu.pl (W.T.); ewa.tabaka@student.umw.edu.pl (E.T.); 3 Division of General and Experimental Pathology, Department of Clinical and Experimental Pathology, Wroclaw Medical University, 50-556 Wroclaw, Poland; 4 Division of Clinical Pathology, Department of Clinical and Experimental Pathology, Wroclaw Medical University, 50-556 Wroclaw, Poland

**Keywords:** diabetes mellitus, diabetic nephropathy, diabetic retinopathy, chronic kidney disease, proteinuria

## Abstract

**Background**: Chronic kidney disease (CKD) affects up to 40% of individuals with diabetes mellitus. Given the fact that CKD in diabetics may result from various non-diabetic renal disorders, kidney biopsy remains essential in cases with atypical clinical presentation. The aim of this study was to assess the prevalence of diabetic nephropathy (DN) and other non-diabetic kidney diseases (NDKD) among diabetic patients who underwent renal biopsy. We also tried to find clinical and laboratory markers predicting the presence of DN in renal tissue. **Methods**: A retrospective analysis of all native renal biopsies in diabetic patients performed between 2010 and 2024 in one European nephrology center. **Results**: The cohort included 115 diabetic patients. DN was diagnosed in 43.5% individuals. Among NDKD cases, vasculitis (8.7%), membranous nephropathy (7.8%), and amyloidosis (7.8%) were most frequent. Compared with the NDKD group, patients with DN were younger, had a longer duration of DM, more often required insulin therapy, more frequently demonstrated diabetic retinopathy (DR) and nephrotic syndrome, and exhibited higher HbA1c levels. In multivariable logistic regression, younger age, need for insulin therapy, and presence of DR were the strongest predictors of DN. **Conclusions**: NDKD is common among DM patients. Patient’s younger age, the need for insulin therapy, and the presence of DR are strong predictive markers for diabetic nephropathy. Renal biopsy remains the most accurate method for diagnosis and should be considered in every case of suspected NDKD.

## 1. Introduction

Diabetes mellitus (DM) is one of the most crucial challenges facing modern medicine. Approximately 589 million adults, aged 20–79 years, are suffering from DM worldwide and it is projected to increase to 853 million by 2050 [1]. It is estimated that about 20–40% of patients with DM will subsequently develop chronic kidney disease (CKD) [2,3].

Current global guidelines suggest avoiding the term “diabetic kidney disease” and use instead “CKD in DM”, emphasizing the fact that not every case of CKD in patient with DM is associated with typical diabetic pathophysiological changes [4,5,6,7]. It may be related to hypertension, dyslipidemia, obesity, renal vascular disease, acute kidney injury (AKI), atherosclerotic glomerulosclerosis, renal ischemia, age-related nephron loss [8], macrovascular disorders, neurogenic bladder, and recurrent urinary tract infections [6].

In clinical practice, diabetic kidney disease is diagnosed when a patient with DM has persistent albuminuria and/or a reduced glomerular filtration rate (GFR) [2,4,9]. The term diabetic nephropathy (DN) refers to diabetes-specific histological changes, observed in renal biopsy, which remains a diagnostic gold standard [2]. The widely used Tervaert histopathological classification distinguishes class I (basement membrane thickening), class II (mesangial proliferation), class III (nodular glomerulosclerosis, so-called Kimmelstiel–Wilson lesions), and class IV (advanced glomerulosclerosis) of DN [10].

Indications for renal biopsy in DM have not been standardized yet and largely depend on the individual center experience [11,12,13]. In most patients, with a typical course of the disease, biopsy is not routinely performed. The most robust indications for biopsy in DM include the sudden onset of nephrotic-range proteinuria, evidence of renal damage in the absence of retinopathy, short DM duration (less than 5 years), presence of active urinary sediment, AKI of unclear etiology, suspected presence of systemic disease, rapid deterioration of renal function, chronically well-controlled blood glucose levels, rapidly rising or high proteinuria, and albuminuria without retinopathy [2,3,13].

The risk of complications associated with renal biopsy in individuals with DM is not higher than in the general population [14]. Furthermore, obesity and diabetic kidney disease appear to be among the factors that reduce the risk of significant post-biopsy bleeding [15]. Contraindications for biopsy include advanced patient’s age, significant comorbidities, antiplatelet and anticoagulant therapy, obesity, and potential additional costs [2]. The benefits of renal biopsy include, above all, the ability to obtain a proper diagnosis, subsequent implementation of treatment, and determining prognosis [2,12,13].

The aim of the present study was to assess the prevalence of DN and other primary or non-diabetic secondary glomerulopathies among patients who underwent renal biopsy. We also try to find clinical and laboratory markers predicting the presence of DN in renal tissue.

## 2. Materials and Methods

All native kidney biopsies, performed between 2010 and 2024, in the Clinical Department of Nephrology, Transplantation Medicine and Internal Diseases in Wrocław, Poland, were retrospectively analyzed. From the whole cohort, data of patients with diagnosed DM were selected for further analysis. The study population consisted of diabetic adult patients (≥18 years) who were hospitalized in the Nephrology Department and underwent renal biopsy. The inclusion criterion was a pre-existing diagnosis of DM before the biopsy was performed. Non-diabetic patients were excluded from the study. The biopsy indications were not standardized and mainly included a non-typical clinical presentation of kidney disease.

The following medical data were taken into account: histopathological diagnoses, demographic information (age, gender), diabetes-related data (type, duration in years, need for insulin therapy, presence of diabetic retinopathy), laboratory test results obtained during hospitalization at the time of biopsy (serum creatinine concentration—sCr, the hemoglobin A1c test—HbA1c, serum albumin concentration—ALB, total serum protein concentration—TP, total cholesterol—TCh, urinary protein expressed as urinary protein to urinary creatinine ratio—UPCR, urinary sediment), and comorbidities (hypertension, atherosclerosis, lupus erythematosus, hematological diseases, heart, lung, liver, and gastrointestinal diseases).

DN and NDKD diagnosis was based on characteristic findings in histopathological examination. DN was diagnosed using the 2010 Tervaert et al. morphological classification [10]. The pathologists were not blinded to clinical data.

Statistical analysis was performed using Statistica ver. 13 software (TIBCO, San Ramon, CA, USA). Data were presented as mean and standard deviation (SD). Comparison between two groups was performed with the use of Student’s *t*-test for parametric variables; for non-parametric variables, a ch2-test was applied. Logistic regression was used to the assess the significance of variables in predicting the final outcome.

## 3. Results

### 3.1. General Characteristic of Population

Among 1293 autologous kidney biopsies, 115 (8.9%) were performed in patients suffering from DM. The majority of diabetic patients suffered from type 2 DM, with only 17% having type 1 DM. Diabetic retinopathy (DR) was found in 13% of patients during ophthalmoscopic evaluation. Nearly half of all patients presented significant, nephrotic-range proteinuria (UPRC > 3.5 g/g). The general characteristics of the patients suffering DM who underwent kidney biopsy are presented in Table 1.

### 3.2. Comparison Between Patients with Biopsy Proven Diabetic Nephropathy and Those with Other Non-Diabetic Kidney Diseases

Patients with biopsy confirmed diabetic nephropathy (DN) were younger, had a longer duration of DM, more frequently needed insulin for disease control, had diabetic retinopathy, and presented with a greater occurrence of nephrotic syndrome than those with other non-diabetic kidney diseases (NDKD). They also had worse glycemic control, as indicated by HbA1c. Patients with type 1 DN usually presented DN in biopsy. A comparison between patients with DN and those with NDKD is presented in Table 2.

### 3.3. Results of Renal Biopsy in Patient with DM

**Figure 1:**
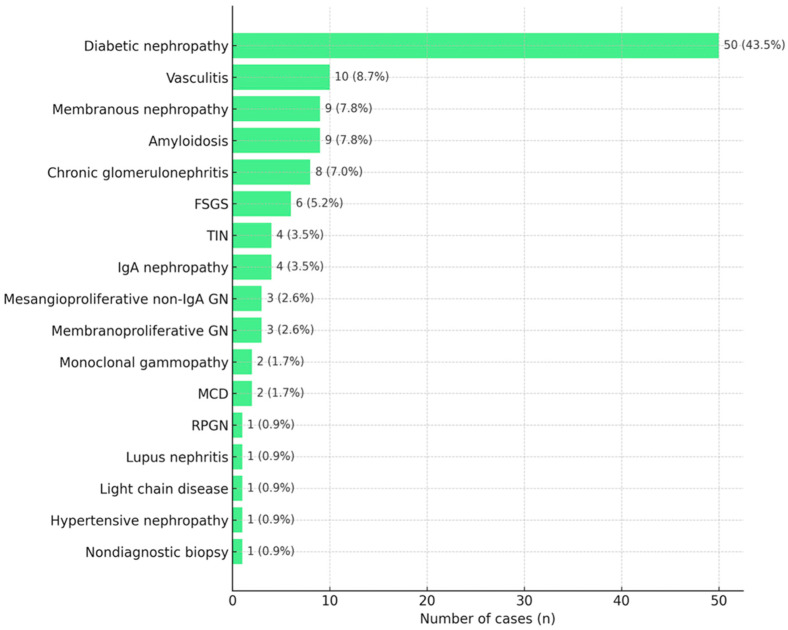
Histopathological diagnoses of renal biopsies in patients with DM.

The most common histopathological diagnosis was DN (43.5%, *n* = 50). Following DN, the next most frequent diagnoses were the following: vasculitis (8.7%, *n* = 10), membranous nephropathy (MN) (7.8%, *n* = 9), amyloidosis (7.8%, *n* = 9), non-specified chronic glomerulonephritis (7%, *n* = 8), focal segmental glomerulosclerosis (FSGS) (5.2%, *n* = 6), IgA nephropathy (3.5%, *n* = 4), tubulointerstitial nephritis (TIN) (3.5%, *n* = 4), membranoproliferative glomerulonephritis (2.6%, *n* = 3), non-IgA mesangioproliferative glomerulonephritis (2.6%, *n* = 3), minimal change disease (MCD) (1.7%, *n* = 2), and monoclonal gammopathy (1.7%, *n* = 2). The rarest diagnoses included light chain disease, hypertensive nephropathy, lupus nephritis, and rapidly progressive glomerulonephritis (RPGN), each accounting for 0.9% (*n* = 1). One biopsy was nondiagnostic (0.9%, *n* = 1). The results are presented in Figure 1.

### 3.4. Factors Predicting Presence of DN in Biopsy

In the logistic regression, the variables were as follows: duration of DM, need for insulin therapy, and presence of DR; NS or HbA1c were selected as having positive impact on DN presence, whereas patients’ age and type 2 DM had a protective impact. Univariate logistic regression analysis results are presented in Table 3.

In the multivariable logistic regression analysis, a set of variables consisting of the patient’s age, the need for insulin therapy, and the presence of DR had the strongest predictive value for diabetic nephropathy, as presented in Table 4.

## 4. Discussion

In our cohort, patients with DM accounted for 8.9% of all patients who underwent kidney biopsy. In comparison to other studies, this percentage varied from 2.3% (in the Chinese population) to 23.5% (in the U.S. population) [16,17,18,19,20]. This might be explained by differences in the incidence of DM across populations as well as differences in biopsy criteria between centers.

Diabetic nephropathy represented 43.5% of all diagnoses among patients suffering from DM. In various studies, this percentage ranged from 6.5% to 94% [13]. Again, these differences are most likely due to small cohorts, geographical and ethnic variations, and different biopsy policies [12].

Among the factors most commonly associated with the diagnosis of DN, there is longer diabetes duration [16,17,21,22,23,24], presence of retinopathy [16,21,22,23,24], and higher HbA1c levels [21,22]. Some studies have also reported associations with nephrotic-range proteinuria [16,17,20,22], insulin therapy [16,23], higher blood pressure [21,22], and chronic lower limb ischemia [16].

A younger age correlated positively with DN in our cohort. Similar findings were reported by Liu et al. [18], Bermejo et al. [19], and Hsieh et al. [25]. Sharma et al. [17] also observed that patients with DN alone were slightly younger than those with NDKD or mixed DN + NDKD. However, age was not a statistically significant predictor of NDKD. In several other studies, no significant age difference was found between DN and NDKD groups [20,23,26,27,28,29]. The age correlation might be explained by the fact that younger patients with DM are more likely to undergo renal biopsy compared to older individuals, as broader biopsy criteria are applied in the younger group considering their longer expected survival and fewer potential contraindications to immunosuppressive therapy compared with older patients.

According to a large study conducted on a cohort of 620 patients by Sharma et al., DM duration of ≥12 years was the strongest predictor of DN, and each additional year of diabetes reduced the probability of NDKD by 5% [17].

In our study, type 2 DM appeared to be associated with a lower prevalence of DN. This finding may be related to the characteristics of the biopsy cohort, including heterogeneous clinical presentations and broader biopsy indications among patients with type 2 DM.

The presence of DR plays a significant role in assessing the likelihood of DN versus NDKD and in determining the indications for kidney biopsy. DR affects approximately 27% of patients with diabetes [30]. Its diagnosing is simple, inexpensive, and non-invasive, which makes it highly useful. A 2019 meta-analysis including 45 studies confirmed that DR is helpful in differentiating DN from NDKD. When the test for DR was negative, the probability of DN decreased to 23%, while a positive result increased it to 70%. Furthermore, the study showed that the severity of DR may not always correlate with the presence of DN [31]. Conversely, another meta-analysis by He et al. reported that proliferative retinopathy may be a highly specific marker of DN [32]. Smaller studies have also shown that DR severity correlated positively with the severity of renal lesions, particularly glomerular changes [22,33,34], and that DR was a risk factor for progression to ESRD [33,34]. Summing up, the absence of DR is a predictor of NDKD, but it does not rule out DN [26].

The data regarding the significance of proteinuria are inconsistent. In some studies, similarly to ours, nephrotic-range proteinuria was associated with a higher risk of DN [16,17,20,22,27,35,36], while in others no such association was demonstrated [23,24,26,37]. Conversely, Bi et al. reported that higher proteinuria was associated with NDKD [28].

Using multiple variable logistic regression, a set of three parameters (patient’s age, the need for insulin therapy, and the presence of DR) was the strongest predictor of DN in renal biopsy in patients with DM. A combination of these three predictors could be a useful tool for distinguishing DN from NDKD and determining indications for renal biopsy. García-Martín et al. [16] developed a predictive model for DN based on a point-scoring system, in which the presence of DR had the highest weight, followed by chronic lower limb ischemia. Depending on the total score, renal biopsy would be indicated or not. Other studies have focused on developing predictive models for NDKD. Zhou et al. [36] proposed a diagnostic model including DMs duration, systolic BP, HbA1c, hematuria, and DR. They demonstrated that the combined evaluation of these factors had higher sensitivity and specificity for diagnosing NDKD than any factor assessed individually. Wong et al. [38] showed that the presence of hematuria or proteinuria ≥ 2 g/day combined with the absence of DR was the strongest indicator of a non-diabetic renal lesion (positive predictive value of 94%).

Among NDKD cases in our cohort, vasculitis was the most common secondary nephropathy, whereas MN was the most frequent primary glomerulopathy. These results differ from other studies, where vasculitis has never been listed among the main diagnoses up until now. In addition, many other studies classified patients not only into DN and NDKD groups, but also mixed DN + NDKD (DN overlapping with NDKD), whereas in our study those patients were not analyzed as a separate group. They were divided into DN and NDKD groups, depending on the dominant type of lesions.

According to a meta-analysis of 48 studies conducted by Fiorentino et al., the most common NDKD overall was IgA nephropathy. It occurred more frequently in Asia than in Europe, America, or Africa. FSGS was more common in Europe than in the USA, and MPGN was more frequent in Asia than in Europe [13]. In contrast, a review by Tong et al., including 40 studies, reported MN as the most frequent NDKD in Asia, Africa, and Europe, and FSGS as the most common in North America and Oceania. However, a limitation of this review was the lack of statistical methods such as meta-analysis [39].

The two most consistent predictors of NDKD are the absence of DR and shorter diabetes duration [19,20,23,25,26,29,40]. Other reported predictors include hematuria [16,24,25,27,28,36], overweight [16,26], lower HbA1c [23,26], lower BP [20,26], lower cholesterol, and higher triglycerides [26], AKI [24].

Hematuria is listed among the indications for biopsy in DM, as its presence may suggest NDKD [2,3]. Dysmorphic erythrocytes > 80% are superior to hematuria in indicating NDKD [41]. However, hematuria may also occur in pure DN, where it can result from ruptured microaneurysms in glomerular capillaries or alterations in the glomerular basement membrane [42]. Okada et al. reported hematuria in 43% of patients with biopsy-proven DN [43]. In other studies, this range was 5–75%, and the variability may be related to the non-uniform definition of hematuria (≥3 or ≥10 RBC/field) [16].

There are several limitations to our study. First, as it was conducted at a single-center European cohort, the results may not be fully representative of other populations or healthcare settings. Moreover, it was retrospective and included kidney biopsies obtained over a 14-year period, which increases the possibility of changes in biopsy indications, pathology interpretation over time, and diabetes management. Furthermore, biopsies were performed only in selected individuals with non-typical clinical presentation that cannot represent the whole population of DM patients with kidney disease. Cases with combined DN and NDKD were not analyzed separately and were classified as DN or NDKD depending on the predominant pathological findings. The inclusion and exclusion criteria for kidney biopsy were not standardized, which may have contributed to selection bias. Despite these limitations, the study provides valuable insights into the studied population.

## 5. Conclusions

To summarize, our study confirms that NDKD is common among DM patients. A patient’s younger age, the need for insulin therapy, and the presence of DR are strong predictive markers for diabetic nephropathy. Renal biopsy remains the most accurate method for diagnosis. It should be considered in every case of suspected NDKD, as a proper diagnosis has a major impact on subsequent therapeutic decisions and further prognosis.

## Figures and Tables

**Table 1:** General characteristics of patients with diabetes.

Variable	Results
Age (years)	59 ± 13
Male (%)	71 (62%)
Duration of DM (years)	11 ± 8
Type 1 DM (%)	20 (17%)
Insulin therapy (%)	52 (45%)
Oral drugs (%)	69 (60%)
Oral drugs + insulin therapy (%)	14 (12%)
DR (%)	15 (13%)
NS (%)	56 (49%)
sCr (mg/dL)	2.17 ± 1.48
HbA1c (%)	6.8 ± 1.3
ALB (g/dL)	2.9 ± 0.8
TP (g/dL)	5.4 ± 1.0
TCh (mg/dL)	244.8 ± 124.3
UPCR (g/g)	5.96 ± 5.39

Abbreviations: ALB—serum albumin concentration; DM—diabetes mellitus; HbA1c—glycated hemoglobin; UPCR—urine protein-to-creatinine ratio; DR—diabetic retinopathy; NS—nephrotic syndrome; sCr—serum creatinine concentration; TCh—total cholesterol; TP—total protein serum concentration.

**Table 2:** Clinical characteristics of patients with DN and NDKD.

Variable	DN (*n* = 50)	NDKD (*n* = 65)	*p*
Age (years)	55 ± 14	62 ± 12	0.002
Male (%)	32 (64%)	39 (60%)	NS
Duration of DM (years)	14 ± 7	7 ± 8	0.006
Type 1 DM (%)	16 (32%)	4 (6.2%)	0.000289
Insulin therapy (%)	34 (68%)	18 (28%)	<0.001
DN (%)	14 (29%)	1 (1.5%)	<0.001
NS (%)	30 (60%)	26 (40%)	0.033
sCr (mg/dL)	1.95 ± 1.38	2.35 ± 1.55	NS
HbA1c (%)	7.3 ± 1.7	6.4 ± 0.7	<0.001
ALB (g/dL)	2.8 ± 0.7	3.0 ± 0.8	NS
TP (g/dL)	5.4 ± 1.1	5.4 ± 1.1	NS
TCh (mg/dL)	250.6 ± 93.9	240.7 ± 142.6	NS
UPCR (g/g)	6.45 ± 6.08	5.57 ± 4.76	NS

Abbreviations: ALB—serum albumin concentration; DM—diabetes mellitus; HbA1c—glycated hemoglobin; UPCR—urine protein-to-creatinine ratio; NS—nephrotic syndrome; sCr—serum creatinine concentration; TCh—total cholesterol; TP—total protein serum concentration.

**Table 3:** Univariable logistic regression in prediction of diabetic nephropathy.

Variable	Estimate	OR	95% CI		*p*-Value
Age [years]	−0.05	0.95	0.92	0.98	0.0035
Presence of type 2 DM [Y/N]	−1.97	0.14	0.04	0.46	0.001
DM duration [years]	0.14	1.15	1.03	1.28	0.011
Insulin therapy [Y/N]	1.71	5.55	2.46	12.52	0.00003
Presence of DR [Y/N]	3.24	25.6	3.16	207.6	0.002
Nephrotic syndrome [Y/N]	0.81	2.25	1.05	4.81	0.035
HbA1c [%]	1.04	2.84	1.51	5.34	0.001

Abbreviations: DM—diabetes mellitus; DR—diabetic retinopathy.

**Table 4:** Multivariate logistic regression in prediction of diabetic nephropathy.

Variable	Estimate	OR	95% CI		*p*-Value
Age [years]	−0.04	0.96	0.92	0.99	0.02
Insulin therapy [Y/N]	1.27	3.56	1.45	8.74	0.006
Presence of DR [Y/N]	2.89	18.01	2.14	151.84	0.008

## Data Availability

The original contributions presented in this study are included in the article. Further inquiries can be directed to the corresponding author.

## Abbreviations

The following abbreviations are used in this manuscript:

AKI	Acute Kidney Injury
ALB	Serum Albumin Concentration
CKD	Chronic Kidney Disease
DM	Diabetes mellitus
DN	Diabetic nephropathy
DR	Diabetic retinopathy
eGFR	Estimated Glomerular Filtration Rate
ESRD	End-Stage Renal Disease
FSGS	Focal Segmental Glomerulonephritis
MCD	Minimal Change Disease
MN	Membranous Nephropathy
MPGN	Membranoproliferative Glomerulonephritis
NDKD	Non-Diabetic Kidney Disease
RPGN	Rapidly Progressive Glomerulonephritis
sCr	Serum Creatinine Concentration
TCh	Total Cholesterol
TIN	Tubulo-Interstitial Nephritis
TP	Total Serum Protein Concentration
UPCR	Urine Protein to Creatinine Ratio
